# Changes in trabecular bone microarchitecture in postmenopausal women with and without type 2 diabetes: a two year longitudinal study

**DOI:** 10.1186/1471-2474-14-114

**Published:** 2013-03-27

**Authors:** Janet M Pritchard, Lora M Giangregorio, Stephanie A Atkinson, Karen A Beattie, Dean Inglis, George Ioannidis, Hertzel Gerstein, Zubin Punthakee, Jonathan D Adachi, Alexandra Papaioannou

**Affiliations:** 1 Faculty of Health Sciences, McMaster University, 1280 Main St West, Hamilton, ON L8S 4K1, Canada; 2 Department of Kinesiology, University of Waterloo, 200 University Avenue West, Waterloo, Ontario N2L 3G1, Canada; 3 Department of Pediatrics, McMaster University, 1280 Main St West, Hamilton, ON L8S 4K1, Canada; 4 Department of Medicine, McMaster University, Charlton Medical Centre, 501-25 Charlton Ave E, Hamilton, ON L8N 1Y2, Canada; 5 Department of Civil Engineering, McMaster University, 1280 Main St West, Hamilton, ON L8S 4K1, Canada; 6 Department of Medicine, McMaster University, 1280 Main St West, Hamilton, ON L8S 4K1, Canada

## Abstract

**Background:**

The risk of experiencing an osteoporotic fracture is greater for adults with type 2 diabetes despite higher than normal bone mineral density (BMD). In addition to BMD, trabecular bone microarchitecture contributes to bone strength, but is not assessed using conventional BMD measurement by dual x-ray absorptiometry (DXA). The aim of this study was to compare two year changes in trabecular bone microarchitecture in women with and without type 2 diabetes.

**Methods:**

We used a 1 Tesla magnetic resonance imaging (MRI) scanner to acquire axial images (resolution 195 μm × 195 μm × 1000 μm) of the distal radius. We report the change in the number and size of trabecular bone holes, bone volume fraction (BVTV), trabecular thickness (Tb.Th), number (Tb.N) and separation (Tb.Sp), endosteal area, nodal and branch density for each group. Lumbar spine and proximal femur BMD were measured with DXA (Hologic, Discovery QDR4500A) at baseline and follow-up. Using a multivariable linear regression model, we evaluated whether the percent change in the trabecular bone microarchitecture variables differed between women with and without type 2 diabetes.

**Results:**

Of the 54 participants at baseline with valid MRI image sets, 37 participants (baseline mean [SD] age, 70.8 [4.4] years) returned for follow-up assessment after 25.4 [1.9] months. Lumbar spine BMD was greater for women with diabetes compared to without diabetes at both baseline and follow-up. After adjustment for ethnicity, women with diabetes had a higher percent increase in number of trabecular bone holes compared to controls (10[1] % versus −7 [2]%, p=0.010), however results were no longer significant after adjustment for multiple comparisons (p=0.090). There were no differences in the change in other trabecular bone microarchitecture variables between groups.

**Conclusion:**

There were no differences in percent change in trabecular bone microarchitecture variables over two years in women with type 2 diabetes compared to women without diabetes. This study provides feasibility data, which will inform future trials assessing change in trabecular bone microarchitecture in women with type 2 diabetes. Larger studies using higher resolution imaging modalities that can assess change in trabecular and cortical bone compartments in women with type 2 diabetes are needed.

## Background

Adults with type 2 diabetes are at 30-70% greater risk of experiencing an osteoporotic fracture than those without type 2 diabetes [1-3], despite normal or higher than normal bone mineral density (BMD) [4]. Various reasons for the greater fracture risk in adults with diabetes have been hypothesized and include medication use [5], accumulation of advanced glycation end-products [6], retinopathy [7], peripheral neuropathy and falls [8]. Bone strength may also be compromised by changes in bone geometry or trabecular bone microarchitecture, which are not reflected in BMD measured with dual x-ray absorptiometry (DXA) [9-11].

Understanding how trabecular bone microarchitecture changes over time may provide insight into the bone fragility observed in adults with type 2 diabetes. In postmenopausal women with type 2 diabetes, we demonstrated that there are larger trabecular bone holes at the distal radius compared to women without diabetes [12], and others have reported that cortical bone is more porous in those with diabetes [13]. Trabecular bone microarchitecture can be modified by osteoporosis treatments [14-16], yet whether there is skeletal response to antiresorptive medication in individuals with diabetes is controversial [17,18].

The primary goal of this study was to explore the hypothesis that postmenopausal women with type 2 diabetes have a greater increase in trabecular bone hole size than women without diabetes when followed over two years. Secondly, we explored whether women with diabetes experience greater increases in the number of trabecular bone holes, trabecular separation (Tb.Sp) and branch density, and greater losses in trabecular bone volume fraction (BVTV), trabecular thickness (Tb.Th), trabecular number (Tb.N) and nodal density than women without diabetes, indicating a loss in bone microarchitectural integrity over time.

## Methods

### Study design and participants

Recruitment for this prospective cohort study occurred between 2008 and 2009. We recruited participants with type 2 diabetes from tertiary care Diabetes Clinics at two sites within Hamilton Health Sciences. Participants without type 2 diabetes were recruited from the community by advertisement. At the time of recruitment, all participants were ≥ 65 years of age, postmenopausal for > 5 years, and those in the diabetes group had been diagnosed with type 2 diabetes for ≥ 5 years [19]. Potential participants were excluded at baseline if they: 1) were taking, or had taken in the past 24 months, any medication known to affect bone, including hormone therapy, calcitonin, selective estrogen receptor modulator, parathyroid hormone, or bisphosphonate; 2) were taking oral glucocorticoids (≥ 2.5 mg/day for ≥ 3 months); or 3) had a diagnosis of a disease known to affect bone (*i.e.,* metastatic cancer in past 5 years, osteogenesis imperfecta, severe renal impairment, hyperparathyroidism, hypoparathyroidism). Participants were asked to complete one study visit as a part of a cross-sectional study published previously [12], and those with valid baseline MRI image sets (absence of motion artifact) were subsequently invited to complete a two year follow-up assessment. This study was approved by the McMaster University Faculty of Health Sciences/Hamilton Health Sciences Research Ethics Board, and all participants provided written informed consent at baseline and follow-up.

### Descriptive variables

Medical history, lifestyle and densitometry data were collected at baseline and follow-up to describe our study participants. Ethnicity was captured and coded as Caucasian or non-Caucasian, which included Native Canadian, Asian, and Caribbean. Ethnicity was used as a covariate in the multivariable linear regression model. A medical history questionnaire was used to assess number of years since menopause, number of years since a diagnosis of type 2 diabetes (if applicable), current medication use, history of major osteoporotic fractures (*i.e.,* non-traumatic fracture of the hip, wrist, vertebral, or proximal humerus) [20] and occurrence of osteoporotic fractures since baseline. The age-adjusted Charlson Index, a global comorbidity index and measure of current health status, was calculated for each participant at baseline and follow-up [21]*.* Physical activity levels were assessed at baseline and at follow-up using a modified Paffenbarger Physical Activity Questionnaire, which quantifies the number of kilocalories (kcal) expended per week based on the number of stairs climbed up, miles walked and participation in recreational activities during a usual week [22]. Each participants’ average supplemental and dietary intake of calcium and vitamin D was estimated at both time-points using a food frequency questionnaire (FFQ) and self-reported supplement intake (including intake from multivitamins) [23]. Anthropometric measurements were collected at baseline and follow-up, and included height, using a wall-mounted stadiometer, weight, obtained from a whole body DXA scan, and waist and hip circumference. A test of grip strength of the dominant hand (Takei T.K.K.5001 Grip A Dynamometer, Takei Scientific Instruments Co. Ltd. Niigata-City, Japan) and a Timed-Up-and-Go (TUG) test were also completed by participants at both time-points. A normative cut-off point of 12.0 seconds was used for TUG test performance [24]. DXA (Hologic, Discovery QDR4500A) scans were acquired to determine BMD at the lumbar spine (L1-L4) and proximal femur (femoral neck and total hip), for descriptive purposes. Whole body DXA scans were performed to estimate body weight and percent body fat. The DXA system’s variability for BMD measurement was 0.315% from the first baseline assessment (September 2008) to the last follow-up assessment (September 2011). Short-term *in vivo* precision was less than 1.70% for BMD measurements [12]. Anonymous DXA scans were analyzed by a certified DXA technician, who was blinded to group membership.

### Magnetic resonance imaging and image analysis

**Figure 1:**
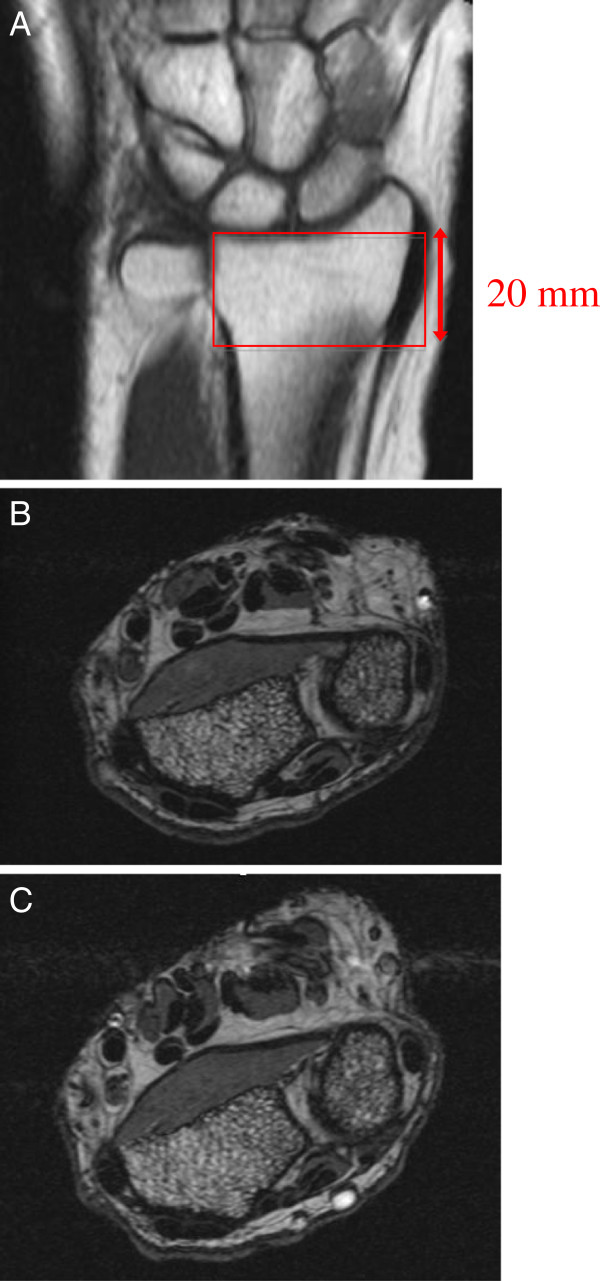
Representative coronal MRI scout scan depicting selection of region of interest for axial slices (A) and matched baseline (B) and follow-up (C) axial MRI images used for analysis.

At baseline and follow-up, each participant’s non-dominant forearm was immobilized in a brace and inserted into the gantry of a 100mm diameter coil in a 1 Tesla peripheral MRI system (OrthOne™, GE Healthcare, United Kingdom). A coronal localizer scan was used to define the region of interest for the axial images (Figure 1). We used a spoiled 3D gradient-echo sequence, which yielded 20 axial slices (195 μm × 195 μm × 1000 μm voxel size) of the distal radius, as previously described (Figure 1) [12]. All scans were performed by the same operator at baseline and follow-up, and a quality control phantom was scanned on a daily basis to ensure system stability.

We used image registration software (Analyze, v.10, Biomedical Imaging Resource at Mayo Clinic, USA) to match baseline and follow-up slices in the axial, sagittal and coronal planes. The first axial slice proximal to the growth plate region of the radius was selected to be the most distal slice in the volume of interest that was analyzed. Given that all participants had at least 8 contiguous slices that matched, 8 matched slices were analyzed per participant for this study. The slices were uploaded for blinded slice-by-slice semi-automatic segmentation using software, previously described [12]. Briefly, binarization of the image separated bone from marrow within the endosteal border of the radius. Binarization reveals holes (*i.e.,* marrow or intertrabecular spaces) of varying sizes within the trabecular bone network. A region growing algorithm was then applied to quantify the number and size of holes in the image [25]. The number of regions grown and the area of the regions grown corresponds to the number of holes in the trabecular bone arrangement and hole size, respectively. Segmentation of trabecular bone within the endosteal boundary of the radius generated nine apparent measures of trabecular bone microarchitecture, including number and size (mm^2^) of trabecular bone holes, endosteal area (mm^2^) and trabecular bone volume fraction (BVTV, %). Following skeletonization, network analysis was performed to assess nodal density (number of nodal points/mm^2^) and branch density (number of branches/mm^2^). A model-independent method was used to estimate apparent trabecular thickness (Tb.Th, mm) and separation (Tb.Sp, mm) [26]. Trabecular number (Tb.N, /mm) was derived using standard histomorphometry formulae (Tb.N= (BVTV)/Tb.Th) [27]. Although Tb.Sp and the size of trabecular bone holes are measures of the same feature of trabecular bone (*i.e.,* marrow or intertrabecular spaces in the trabecular bone network), these indices are different because they are derived using different techniques. Trabecular separation is a one-dimensional measurement and is derived using the three-dimensional distance transform technique, where marrow spaces are filled with maximal spheres and Tb.Sp is computed as the mean diameter of the spheres [26,28]. Trabecular bone hole size is a two-dimensional measurement of hole area and is computed as the mean area of holes grown [25]. The baseline comparison of trabecular bone microarchitecture between women with and without diabetes has been published and reflects the analysis of the central 6 MRI slices [12]. The root mean square coefficient of variation (RMSCV%) ranged from 1.10% to 4.90% and intraclass correlation coefficient ranged from 0.83 to 0.99 for the assessment of trabecular bone microarchitecture variables [12].

### Statistical analyses

The Kolmogorov-Smirnov test was used to confirm normal distribution of all variables, therefore descriptive data are presented as mean (standard deviation, SD) for continuous variables, and frequency (%) for categorical variables. Between-group differences in descriptive variables at baseline and follow-up were determined using an unpaired Student’s *t-*test or Chi-square test. For the assessment of internal validity, an unpaired Student’s *t-*test was employed to compare baseline descriptive variables and trabecular bone microarchitecture variables for the participants who dropped out and returned for the follow-up visit. The absolute change in trabecular bone microarchitecture was calculated as follows: follow-up measurement−baseline measurement.

Mulitvariable linear regression was applied to answer the primary question of whether percent change in trabecular bone microarchitecture differed in women with diabetes compared to women without diabetes. The nine dependent variables were: percent change (absolute change/baseline measurement x 100%) in size and number of trabecular bone holes, endosteal area, BVTV, Tb.Th, Tb.Sp, Tb.N, branch density and nodal density. Inclusion of ethnicity in the model was based on previous literature suggesting that ethnicity influences BMD [29], and on the statistical principle that a covariate is significantly related to the primary dependent variable (percent change in trabecular bone hole size) [30]. Pearson correlation analysis revealed that ethnicity was related to the primary dependent variable (r= −0.364, p=0.038). The Holm’s test for multiple comparisons was used for the comparison of percent changes in secondary trabecular bone microarchitecture variables between groups [31]. The adjusted means and SD are presented. The criterion for statistical significance was set at alpha < 0.05. All analyses were performed with SPSS version 20 (IBM Corporation, Somers, USA).

## Results

### Study participants

**Figure 2:**
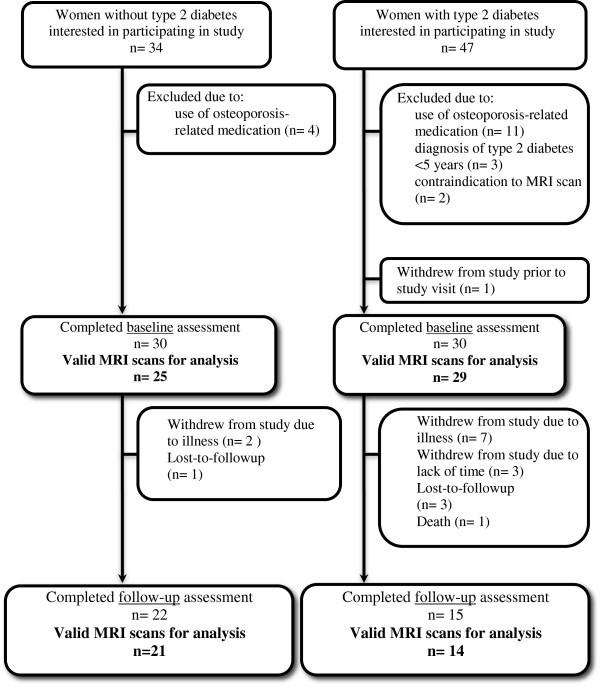
Path outlining study participant recruitment, enrollment and follow-up from baseline to follow-up assessment.

The descriptive characteristics of all study participants who completed baseline and follow-up assessments are shown in Table 1. At baseline, 6 MRI image sets were unacceptable for analysis due to motion artifact. Of the 54 participants with valid baseline MRI scans, 15/29 (52%) participants with type 2 diabetes and 22/25 (88%) participants without diabetes returned for the follow-up assessment (Figure 2).

**Table 1:** Descriptive characteristics of all study participants who were enrolled at baseline and follow-up

	**Baseline**			**Follow-up**		
	**Women with diabetes n= 30**	**Controls n= 30**	**Difference between groups *p-value***	**Women with diabetes n= 15**	**Controls n= 22**	**Difference between groups *p-value***
Age, years	71.1 (4.8)	70.7 (4.9)	0.816	73.9 (3.6)	72.5 (4.9)	0.324
Caucasian, n (%)	23 (79.3)	30 (100.0)	0.017*	12 (80)	22 (100.0)	0.009*
History of osteoporotic fracture^a^						
Since age 40 years, n (%)	5 (17.7)	6 (20.0)	0.738	-	-	-
Since baseline assessment, n (%)	-	-	-	2 (13.3)	1 (4.5)	0.315
BMI, kg/m^2^	34.6 (7.6)	27.9 (5.5)	<0.001*	36.1 (5.7)	27.9 (4.4)	<0.001*
Waist:hip Ratio	0.89 (0.07)	0.83 (0.06)	0.002*	0.90 (0.05)	0.83 (0.06)	<0.001*
Body fat percentage, %	40.3 (6.1)	37.2 (6.5)	0.056	41.8 (9.5)	39.1 (4.2)	0.256
Time since menopause, years	22 (7)	22 (8)	0.841	24 (5)	23 (7)	0.656
Number of prescribed medications	6.6 (3.5)	1.9 (2.2)	<0.001*	8.1 (3.0)	2.4 (2.5)	<0.001*
Age-adjusted Charlson Index	4.3 (1.5)	0.1 (0.6)	<0.001*	4.5 (1.2)	0.1 (0.6)	<0.001*
Total calcium intake, mg/day	1594 (696)	2062 (590)	0.007*	1679 (890)	2019 (639)	0.697
Supplemental, mg/day	446 (481)	678 (482)	0.070	377 (480)	603 (427)	0.138
Dietary, mg/day	1148 (564)	1397 (335)	0.054	1345 (660)	1241 (473)	0.565
Total vitamin D intake, IU/day	806 (622)	1177 (912)	0.073	1316 (828)	1488 (875)	0.562
Supplemental, IU/day	626 (573)	982 (921)	0.080	993 (822)	1285 (866)	0.308
Dietary, IU/day	179 (142)	195 (130)	0.644	252 (124)	218 (155)	0.495
Weekly energy expenditure, kcal/week	1984 (2428)	2584 (2203)	0.333	959 (1129)	2255 (1443)	0.005*
TUG Test, seconds	12.8 (4.0)	9.4 (2.7)	<0.001*	14.4 (4.4)	10.0 (3.4)	<0.001*
TUG test >12 seconds, n (%)	11 (44.0)	4 (13.3)	0.011*	7 (46.6)	2 (9.1)	0.005*
Grip strength, kg	18.8 (4.8)	21.7 (6.3)	0.058	16.3 (5.0)	20.2 (6.1)	0.048*
Bone density measurements						
Lumbar spine, g/cm^2^	1.07 (0.15)	0.97 (0.19)	0.025*	1.11 (0.15)	0.99 (0.15)	0.022*
Lumbar spine T-score	0.15 (1.40)	-0.61 (1.66)	0.038*	0.47 (1.27)	−0.51 (1.34)	0.034*
Femoral neck, g/cm^2^	0.73 (0.11)	0.69 (0.09)	0.254	0.73 (0.11)	0.69 (0.09)	0.254
Femoral neck T-score	−1.11 (1.02)	1.40 (0.89)	0.288	−1.14 (0.68)	−1.31 (0.88)	0.524
Total hip, g/cm^2^	0.87 (0.12)	0.86 (0.11)	0.639	0.88 (0.12)	0.87 (0.10)	0.759
Total hip T-score	−0.58 (0.99)	-0.70 (0.95)	0.657	−0.54 (0.72)	−0.59 (0.87)	0.853

Values are mean (SD), unless indicated. * indicates significant between-group differences at p-value <0.05.

^a^Atraumatic osteoporotic fracture includes hip, wrist, spine or proximal humerus fracture.

*Abbreviations*: body mass index, BMI; timed-up-and-go, TUG.

The average time between baseline and follow-up visits was 25.4 (1.9) months. At follow-up, women with type 2 diabetes had a diagnosis of diabetes for 18.8 (9.7) years, and the majority of participants (12/15 [80.0%]) were taking insulin or insulin in combination with another glucose-lowering intervention. The remaining participants were either taking metformin (2/15 [13.3%]) or no medication (1/15 [6.7%]). At baseline and follow-up, the group of women with diabetes was comprised of fewer Caucasians with a greater BMI who were prescribed more medications. Lumbar spine BMD was also greater for women with diabetes at both time-points (Table 1).

### Differences between study participants and drop-outs

The participants who dropped out of the study were not different from those who returned for the follow-up visit, regarding the majority of descriptive characteristics presented in Table 1. The only exception was for percent body fat in the women without diabetes, which was greater for those that returned for follow-up compared to those who dropped out (39.1 [4.2]% versus 31.1 [9.0]%, p=0.003) (remaining data not shown). Regarding baseline microarchitectural differences, trabecular bone holes were larger (2.51 [0.31]mm^2^ versus 2.14 [0.43]mm^2^, p=0.042), BVTV was lower (46.9 [0.3]% versus 47.6 [0.9]%, p=0.017), and branch density was greater (0.48 [0.03]/mm^2^ versus 0.41 [0.06]/mm^2^, p=0.003) in women with diabetes who dropped out of the study compared to those who returned for the follow-up visit. In women without diabetes, the number of trabecular bone holes was greater (92 [12] holes versus 72 [15] holes, p=0.031) and hole size smaller (1.75 [0.17]mm^2^ versus 2.06 [0.42]mm^2^, p=0.030) in those who dropped out of the study compared to those who returned for follow-up.

### Between-group differences in change in trabecular bone microarchitecture

Two MRI scans were considered unacceptable for analysis due to motion artifact, resulting in 14 valid image sets for the type 2 diabetes group and 21 valid image sets for the control group. Unadjusted baseline, follow-up and absolute change in trabecular bone microarchitecture variables are summarized in Table 2 for the participants who had valid MRI image sets at baseline and follow-up. Table 3 shows the adjusted percent changes in trabecular bone microarchitecture variables for both groups. Women with diabetes had a significantly higher percent increase in the number of trabecular bone holes as compared to women without diabetes (10 [1]% versus −7 [2]%, p=0.010) and there were no differences between groups in the change in trabecular bone hole size (−4.15 [4.88]% versus 5.03 [3.90]%, p=0.172) or in other trabecular bone microarchitecture variables (Table 3). After adjustment for multiple comparisons, there were no between-group differences in percent change in trabecular bone microarchitecture variables.

**Table 2:** Unadjusted trabecular bone microarchitecture measures for participants with valid MRI images who completed both baseline and follow-up assessments

	**Women with type 2 diabetes**			**Controls**			
	**Baseline n= 14**	**Follow-up n= 14**	**Absolute change from baseline**	**Baseline n= 21**	**Follow-up n= 21**	**Absolute change from baseline**	**Between group difference *p-value***
Hole size, mm^2^	2.10 (0.47)	2.04 (0.37)	−0.06 (0.48)	2.06 (0.42)	2.08 (0.45)	0.03 (0.32)	0.513
Number of holes	68 (17)	69 (13)	1 (15)	72 (15)	68 (18)	−4 (12)	0.283
Endosteal area, mm^2^	260.7 (51.1)	264.8 (56.9)	4.0 (39.9)	273.2 (58.4)	258.3 (48.9)	−14.9 (39.6)	0.939
BVTV, %	47.7 (1.0)	47.9 (0.8)	0.2 (0.9)	47.7 (1.2)	47.8 (1.0)	0.1 (0.7)	0.759
Tb.Th, mm	0.52 (0.01)	0.51 (0.01)	0 (0.01)	0.51 (0.01)	0.51 (0.01)	0 (0.01)	0.549
Tb.Sp, mm	0.55 (0.01)	0.54 (0.01)	0 (0.01)	0.54 (0.02)	0.54 (0.02)	0 (0.01)	0.322
Tb.N,/mm	0.92 (0.03)	0.93 (0.02)	0.01 (0.02)	0.93 (0.03)	0.93 (0.03)	0 (0.01)	0.362
Nodal density, /mm^2^	0.16 (0.01)	0.16 (0.01)	0 (0.01)	0.16 (0.01)	0.15 (0.01)	0 (0.01)	0.574
Branch density, /mm^2^	0.41 (0.06)	0.42 (0.05)	0.01 (0.05)	0.41 (0.05)	0.42 (0.06)	0.01 (0.05)	0.940

Values are mean (SD). Between group difference for absolute change in trabecular bone microarchitecture measures for women with and without type 2 diabetes.

*Abbreviations:* BVTV, bone volume fraction; Tb.Th, trabecular thickness; Tb.Sp, trabecular separation; Tb.N, trabecular number.

**Table 3:** Adjusted percent changes over two years in trabecular bone microarchitecture variables for women with and without type 2 diabetes

	**Women with type 2 diabetes n=14**	**Controls n=21**	**Between group difference *p*-value**	**Holm’s adjusted *p*-value**
Hole size	−4.1 (4.9)	5.0 (3.9)	0.172	
Number of holes	10.1 (5.2)	−7.1 (4.4)	0.010	0.090
Endosteal area	2.8 (4.4)	−4.5 (3.5)	0.225	1.00
BVTV	0.7 (0.4)	0.1 (0.4)	0.263	1.00
Tb.Th	−0.6 (0.5)	−0.3 (0.4)	0.661	1.00
Tb.Sp	−1.0 (0.4)	−0.2 (0.3)	0.206	1.00
Tb.N	1.3 (0.5)	0.3 (0.4)	0.119	0.952
Nodal density	−0.8 (1.8)	−3.8 (1.4)	0.221	1.00
Branch density	0.5 (3.6)	3.3 (2.9)	0.566	1.00

Values are mean (SD). Multivariate analyses adjusted for ethnicity.

## Discussion

Results of our study revealed that over two years of follow-up, changes in trabecular bone microarchitecture are not different in women with and without type 2 diabetes. This is the first study to explore longitudinal changes in trabecular bone microarchitecture in women with type 2 diabetes. Our study provides important feasibility data which should be considered when planning and conducting subsequent longitudinal studies on trabecular bone microarchitecture change in older women with type 2 diabetes. The women with type 2 diabetes who did not attend the follow-up study visit had a different baseline trabecular bone microarchitecture phenotype than those who did attend the follow-up study visit. We demonstrated that for participants with type 2 diabetes who dropped out of the study, trabecular bone was less intact (*i.e.,* larger trabecular bone holes, lower BVTV, greater branch density). Thus, our preliminary internal validity data suggests that it may be important to increase the frequency of study visits (*i.e.,* annually or semi-annually) for older participants with type 2 diabetes and multiple comorbidities in order to retain participants and obtain data that is more generalizable to patients with type 2 diabetes of varying disease severity. Subsequent studies with adequate follow-up are needed to verify our observations. In particular, higher resolution imaging systems should be used to examine changes in trabecular bone microarchitecture relative to changes in cortical bone structure (*i.e.,* cortical thickness, porosity), as cross-sectional studies suggest that cortical bone is compromised in women with type 2 diabetes [13].

Skeletal change in adults with type 2 diabetes has been limited to the description of BMD change; however, whether individuals with diabetes lose bone at a faster rate than non-diabetics is unclear [32-34]. Younger women with diabetes [33] and women with newly diagnosed diabetes [34] experienced greater losses in hip BMD, whereas in women with diabetes for more than twelve years [32] and in postmenopausal women [34], no differences in the rate of BMD loss have been reported. It is possible that the greatest losses in bone occur during the years surrounding the diagnosis of type 2 diabetes when the likelihood of hyperglycemia, hypercalcuria, and generation of reactive oxygen species (ROS) is high [35-37]. The negative impact of hyperglycemia and ROS on osteoblasts has been demonstrated in vitro [38,39], and is a potential mechanism causing diabetic bone fragility [40]. Variability exists in the concentration of these factors in adults with type 2 diabetes, depending on duration and control of diabetes [35]. This may explain the discrepancy in BMD change in women with diabetes, and supports our finding of no difference in the change in trabecular bone microarchitecture variables in older postmenopausal women with long-standing type 2 diabetes given that the average length of time since diabetes diagnosis in our study was over 18 years. Weight loss is another factor involved in proximal femur bone loss in adults with type 2 diabetes [41,42]. In a large prospective study, Caucasian women with type 2 diabetes lost more femoral neck BMD over 4 years than Caucasian women without type 2 diabetes, and weight loss was an important mediator of this relationship [41]. The mechanism involved is likely related to the reduction in skeletal loading with weight loss [43]. In our study, weight was approximately stable over the two years in both groups, and weight change was not related to the change in trabecular bone hole size (data not shown). Further, we assessed trabecular bone at a non-weightbearing site, which is unlikely to be impacted by weight change.

In studies with similar sample sizes to our study, non-diabetic women taking alendronate [44] and estrogen supplementation [16] experienced no change in some microarchitectural variables assessed with MRI at the radius. Similarly, nasal calcitonin does not change trabecular bone microarchitecture at the more distal sites of the radius, but does preserve microarchitecural quality at proximal radius sites [14]. We speculate that losses in trabecular bone microarchitecture at more proximal sites might be apparent in women with newly diagnosed type 2 diabetes, which should be investigated in the future. While measures of trabecular bone microarchitecture can be assessed using MRI, peripheral quantitative computed tomography (pQCT) and high-resolution pQCT (HR-pQCT), the superior signal to noise ratio with MRI allows for improved differentiation between bone and marrow [45-47]. Studies have however shown moderate to strong correlations between measures derived by pQCT or HR-pQCT and MRI [10,48].

There were several study limitations. First, our sample size was small as approximately 50% of participants with type 2 diabetes either dropped out of the study, were lost to follow-up or died after the baseline assessment. To assess the internal validity of the study, we compared the baseline descriptive characteristics and trabecular bone microarchitecture variables for the participants who dropped out to those who remained in the study. In women with diabetes who dropped out, the trabecular bone microarchitecture appeared less intact, and in women without diabetes who dropped out, the trabecular bone network appeared to have more holes compared to those who returned. It is possible that the individuals who dropped out had more comorbidities that were not assessed in this study. For example, subclinical peripheral arterial disease, which we did not assess, has been linked to reduced bone mineral content [49] and to osteoporotic fractures in adults with type 2 diabetes [3]. Due to the large number of participants who did not return for follow-up assessment, our results may have been biased towards not detecting a difference in trabecular bone microarchitectural changes, given the baseline differences between those who dropped out and returned to complete the study. Our analysis should be considered exploratory and will need to be confirmed in larger studies. We were also unable to accurately capture diabetes-related complications in this study, however future studies should consider the role of comorbidities, particularly neuropathy and nephropathy [41] when examining bone loss in diabetics. While multivariable linear regression models were used to account for the differences in ethnicity between women with diabetes and controls, the study would have been strengthened if participants were matched based on ethnicity. In addition, the resolution of the images acquired with our 1 Tesla MRI system restricts our analyses to trabecular bone, and is not appropriate for the assessment of distal radius cortical bone. A 1 Telsa MRI system is limited by signal strength, although we attempted to optimize signal strength by using a small (100 mm diameter) radio-frequency coil to enhance the signal-to-noise ratio. The lack of overall microarchitectural change may have been due to the lower signal strength of the 1 Tesla MRI system and it’s limited ability to detect small changes in microarchitecture, such as the thickness of trabeculae. Future studies should explore longitudinal changes in trabecular bone microarchitecture using 1.5, 3 or 7 Tesla systems which have superior signal-to-noise ratio and higher image resolution. Finally, no prospective data were available at study inception on the change in the size or number of trabecular bone holes, therefore we were unable to estimate an ideal sample size required at follow-up to capture differences in these key variables. Given our study limitations, larger studies with more complete follow-up, the ability to look at multiple study outcomes and those that assess potential confounders (*i.e.,* diabetes related complications) are needed prior to making definitive conclusions about the lack of change in trabecular bone microarchitecture in women with type 2 diabetes.

## Conclusion

This study provides early evidence suggesting that trabecular bone microarchitecture change is not different in women with type 2 diabetes compared to women without type 2 diabetes. Understanding whether microarchitectural adaptations of the trabecular and cortical bone with type 2 diabetes are distinctly different from age-related changes would inform future research and fracture prevention strategies in adults with type 2 diabetes.

## Competing interest

The author(s) declare that they have no competing interests.

## Authors’ contributions

JMP, LMG, SAA, KAB, HP, ZP, JDA and AP were involved in the study conception, design and interpretation of results. JMP conducted study visits, collected data and drafted the manuscript. DI was involved in MRI protocol development, software design and data collection. GI contributed to the study design and conducted statistical analyses. All authors edited, read and approved the final manuscript.

## Pre-publication history

The pre-publication history for this paper can be accessed here:

http://www.biomedcentral.com/1471-2474/14/114/prepub
